# Linking Epigenetics, Metabolism and Cancer: Lessons From SIRT6

**DOI:** 10.1093/geroni/igaa057.2651

**Published:** 2020-12-16

**Authors:** Raul Mostoslavsky

**Affiliations:** Mass General Cancer Center, Boston, Massachusetts, United States

## Abstract

In recent years, chromatin regulators have emerged as key modulators in cancer. We discovered that the mammalian histone deacetylase SIRT6 is a key chromatin factor, modulating expression of metabolic, developmental, and ribosomal protein genes. In recent studies, we identified a loss of function mutation in SIRT6 behind a human syndrome of perinatal lethality. In the context of cancer, we found SIRT6 to act as a robust tumor suppressor, by modulating glucose metabolism. As Otto Warburg described decades ago, cancer cells exhibit glycolytic metabolism, where pyruvate, instead of contributing to ATP production in the mitochondria, is converted to lactate even under normoxia conditions. We found SIRT6 as the first chromatin factor in charge of suppressing the Warburg effect in colon and skin cancer. At the cellular level, SIRT6 inactivation leads to increased cellular glucose uptake, higher lactate production and decreased mitochondrial activity. Our results indicate that SIRT6 directly regulates expression of several key glycolytic and ribosomal genes, co-repressing Hif1a and Myc, respectively, and acting as a histone H3 lysine9 (H3K9) and lysine 56 (H3K56) deacetylase. Notably, we recently identified SIRT6 as the first deacetylase to specifically inhibit transcriptional elongation, rather than initiation, in its targets. Strikingly, we determined in new studies that such glycolytic switch provides an advantage even at the early initiating cancer stem cells stage, in what we identified as the cell-of-origin for the Warburg effect. In addition, we found SIRT6 to act as a robust tumor suppressor in the context of pancreatic cancer. However, in this case, SIRT6 did not influence metabolism, but rather silenced expression of the developmental gene Lin28b, in this way protecting against aggressive undifferentiated pancreatic adenocarcinoma. Our studies highlight the important role epigenetic factors, such as SIRT6, play in protecting against tumor progression by providing “epigenetic plasticity”, inhibiting adaptive responses in transformed cells.

